# Evaluating the contact anatomy and contact bone volume of spinal screws using a novel drilled surface image

**DOI:** 10.1371/journal.pone.0282737

**Published:** 2023-04-10

**Authors:** Yun-Xuan Tang, Shin-Lei Peng, Yi-Wen Chen, Hsiang-Ming Huang, Cheng-Ting Shih

**Affiliations:** 1 Department of Radiology, Shin Kong Wu Ho-Su Memorial Hospital, Taipei, Taiwan; 2 Department of Medical Imaging and Radiological Technology, Yuanpei University of Medical Technology, Hsinchu, Taiwan; 3 Department of Biomedical Imaging and Radiological Science, China Medical University, Taichung, Taiwan; 4 Graduate Institute of Biomedical Sciences, China Medical University, Taichung, Taiwan; 5 x-Dimension Center for Medical Research and Translation, China Medical University Hospital, Taichung, Taiwan; 6 3D Printing Medical Research Institute, Asia University, Taichung, Taiwan; 7 Department of Neurosurgery, China Medical University Hsinchu Hospital, Hsinchu, Taiwan; AIIMS: All India Institute of Medical Sciences, INDIA

## Abstract

Intraoperative navigation systems have been widely applied in spinal fusion surgery to improve the implantation accuracy of spinal screws using orthogonal tomographic and surface-rendering imaging. However, these images contain limited anatomical information and no information on bone volume contact by the implanted screw, which has been proven to affect the stability of implanted screws. This study proposed a novel drilled surface imaging technique that displays anatomical integration properties to calculate the contact bone volume (CBV) of the screws implanted along an implantation trajectory. A cylinder was used to represent the area traversed by the screws, which was manually rotated and translated to a predetermined implantation trajectory according to a vertebra model obtained using computed tomography (CT) image volumes. The drilled surface image was reconstructed by interpolating the CT numbers at the predefined sampling points on the cylinder surface. The anatomical integration property and CBV of the screw implanted along the transpedicular trajectory (TT) and cortical bone trajectory (CBT) were evaluated and compared. The drilled surface image fully revealed the contact anatomical structure of the screw under the trajectories, improving the understanding of the anatomical integration of the screw and surrounding tissues. On average, the CBV of the CBT was 30% greater than that of the TT. The proposed drilled surface image may be applied in preoperative planning and integrated into intraoperative navigation systems to evaluate the anatomical integration and degree of bone contact of the screw implanted along a trajectory.

## Introduction

The spine supports the body, controls activity, and protects nerve tissue. Disc degeneration or atrophy in patients due to external trauma, bone fractures, bone diseases, or degenerative arthritis from age reduce intervertebral connectivity and cause slipped discs [1, 2]. Slipped discs apply pressure to the surrounding tissue and nerves, cause patients to lose strength and function in their lower limbs, and may even cause irreversible neurological injury [3, 4]. The curvature of the spine may also be abnormal due to disease and sports injuries. Especially in patients with congenital scoliosis and torsion, the deformed spine could be severely curved and misaligned [5, 6].

Spinal fusion surgery is a common procedure for correcting loose or malformed spines that uses vertebral screw fixation to integrate and correct multiple vertebrae. To increase implant accuracy, prevent damage to the surrounding nerves and blood vessels, and shorten the procedure time, an intraoperative navigation system with intraoperative computed tomography (CT) [7–9] or O-arm CT is widely used [10–13]. These techniques produce sectional images of a patient’s current spatial position. The outline and spatial position of the implements and implants used in the procedure are captured using external lasers or infrared positioning devices. After the spatial alignment of completed images and implements, physicians can operate on the implements to compare inflicted areas, and the navigation system can immediately generate orthogonal tomographies of corresponding positions by using surgical implements and three-dimensional (3D) surface rendering. Therefore, operating physicians can observe these images to determine whether screw implantation paths and particular directions are appropriate. However, stereoscopic models generated using the navigation system only reveal bone surface information; operating physicians still must combine coronal, sagittal, and axial two-dimensional sections to determine the corresponding locations of screws in the spine and the areas that the screws are expected to traverse after implantation. Thus, the accuracy of screw implantation still relies on operating physicians’ experience and judgment. Carelessness may lead to unexpected screw paths and damage to the surrounding nerves, blood vessels, and tissues. Moreover, visual assistance from stereoscopic models of 3D surface rendering may only display structural surfaces of grayscale values and cannot provide information regarding the internal anatomy.

The mechanical stability of screw implantation determines the steadiness of pedicle screw fixation surgery. However, once screws have loosened, the original spondylolisthesis reoccurs, and loosened screws may damage the spine, surrounding blood vessels, and even the nerves [14]. The stability of implanted screws relies on the bite force of screws when contacting rigid structures in the vertebral body. Crucially, the spine is mainly composed of a surface cortical bone layer and trabeculae and bone marrow. The rigid structures encountered by implanted screws are the cortical bone layer and trabeculae. Therefore, the stability of screws in the vertebral body is determined by the skeletal structure encountered. Numerous studies have investigated the effect of bone quality on screw stability. Cortical bone thickness and trabecular structure density may account for 90% of screw mechanical stability [15]. Furthermore, implantation paths differing from the traditional transpedicular trajectory (TT) that provide higher stability have been proposed, including a vertical and cortical bone trajectory (CBT) [16–18]. Reports have indicated that screws have higher stability when patients have higher bone mineral density and bone volume fraction and that screws encounter more bone structures in their path [19–22]. Numerous studies have used CT imaging to measure the implantation depth or angle of spinal screws under a specific trajectory for morphological evaluation [23, 24] and to calculate the mean CT number or bone mineral density along the trajectory for screw–bone contact evaluation [25–27]. Radiography has also been used to assess the integration of spine and screw [28]. However, CT images are two-dimensional sectional images that only display partial information, and radiography provides projection images that lack depth information and cannot fully represent screw–bone contact. No effective methods are currently available to assess the bone volume encountered in screw paths that are suitable for integration with navigation systems.

This study proposed a novel screw-drilled surface imaging technique to assist in spinal fusion surgery. After acquiring patient CT images and capturing the spine geometry, the surgeon defined the screw path and reconstructed the drilled surface image from the CT images. The anatomical structure the screw contacted in the defined path could be comprehensively determined using this image alone. The drilled surface image indicated the CT number, which could be converted into a bone-volume distribution map to aid in calculating the bone volume encountered in the path. In this study, the proposed drilled surface imaging was performed on patients undergoing clinical spinal fusion surgery, and drilled surface images of the fourth lumbar vertebrae were generated to compare the contacted bone volume in the TT and CBT.

## Materials and methods

### Reconstruction of drilled surface images

**Fig 1:**
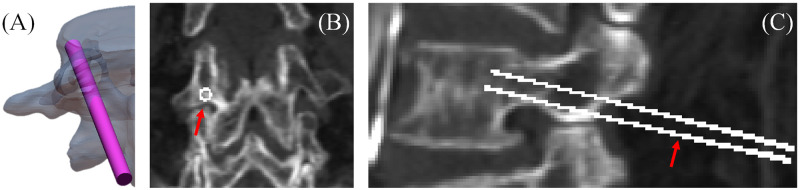
(A) Cylinder placed along the CBT path. (B) coronal and (C) sagittal CT images of a lumbar spine labeled with sampling points redefined from the coordinates of postdisplacement cylindrical mesh model vertexes.

In this study, the drilled surface image was reconstructed using the screw path identified using CT image volume. First, threshold segmentation was used to capture the spine from the CT image volume, and then a mesh model of the segmented spine was constructed. The mesh vertex coordinates remained in the same coordinate system as the CT image volume voxel coordinates. A cylindrical mesh model with the same 4.5-mm diameter as the actual spinal screws was drawn to define the area through which the screw traverses. The cylinder was then positioned in the TT and CBT paths using translation and rotation, as shown in Fig 1A, and the displacement and rotation angles of the cylinder were recorded. On the basis of the postdisplacement cylindrical mesh model vertex coordinates, the CT numbers of these coordinates were interpolated from the CT image volume, and the drilled surface image was formed by mapping the average CT values of vertices forming the same mesh surface onto the mesh surface. After unfolding the cylindrical mesh, the drilled surface image was reconstructed. However, the image was drawn according to the mesh shape, not a regular image matrix, and thus, subsequent processing and display were difficult to apply. Therefore, the cylinder posttranslation and postrotation starting coordinates were used to redefine the regular sampling points in this study with 0.1 mm spacing. The CT numbers of these coordinates were then interpolated from the CT image volume. Fig 1B and 1C present the redefined sampling points labeled on the CT images for the CT number interpolation. Finally, the calculation results were arranged in a matrix according to the relationships between sampling points, which generated a rules matrix drilled surface image.

### Calculation of CBVs from drilled surface images

Because the drilled surface image was reconstructed from the CT image volume, its grayscale values were represented as CT numbers. Algorithms converting CT numbers into bone volume have been widely studied and discussed. In this study, the CT numbers in the drilled surface image were converted to bone volumes using the two-compartment model, which can accurately calculate bone volume from CT images by matching those calculated from micro-CT images through the threshold method; this indicates the absolute bone mass [29]. The calculations are summarized subsequently. Given that bones are predominantly composed of bone marrow and cortical bone, in addition to the Bragg rule and formula defining CT values, the relationship between bone volume and CT number in each voxel was described using the following equation:

$$BV=\frac{CTN_{B.}-CTN_{Meq.}}{CTN_{Beq.}-CTN_{Meq.}}\times v,$$ (1)

where *CTN*_*Meq*._, *CTN*_*Beq*._, and *CTN*_*B*._ represent the CT numbers of the bone marrow–equivalent material, cortical bone–equivalent material, and bones under test, respectively, and *v* represents the voxel size. In this study, the CT numbers of the bone marrow and cortical bone–equivalent materials were obtained from scanning and measuring water and an electron density calibration phantom (RMI 467, Gammex, Middleton, WI, USA). Through the use of Eq 1, the drilled surface image can be converted into a contact bone volume map (CBV map), and then the total contact bone volume (total CBV) can be calculated along the entire path.

### Comparison of TT and CBT in contact anatomy and CBVs

The proposed drilled surface image and CBV map were applied to compare the contact anatomy and CBV of the spinal screw under both TT and CBT paths using patient CT images. With approval from the Institutional Review Board (CMUH107-REC3-087), the presurgery CT images of patients undergoing spinal fusion surgery were retrospectively collected. After excluding patients with lumbar vertebral fractures, prior instrumentation, deformities, or intensive image artifacts presented in their CT images [24, 27], 55 patients aged 26–100 years (mean age, 62.6 years) were included. The CT images were acquired using a multidetector row CT scanner (Aquilion 64, Toshiba Medical Systems, Tochigi, Japan) in a helical model with a tube voltage of 120 kVp and a tube current of auto exposure control. The images were reconstructed into 512 × 512 matrices with 1-mm slice thickness. Then, drilled surface images were reconstructed from the bilateral vertebral TT and CBT implantation paths defined by a neurosurgeon. The average and total CBV and the CBV per unit implanted depth were calculated using the images. In the statistical analysis, the association between a patient’s age and CBV was assessed using correlation analysis and the Pearson correlation coefficient. The effects of the TT and CBT paths on CBV were assessed using the paired-samples *t*-test. All analyses were performed using MedCalc statistical software (version 15.8; MedCalc Software, Ostend, Belgium). Any *p*-value calculated as <0.05 indicated statistical significance.

## Results

**Fig 2:**
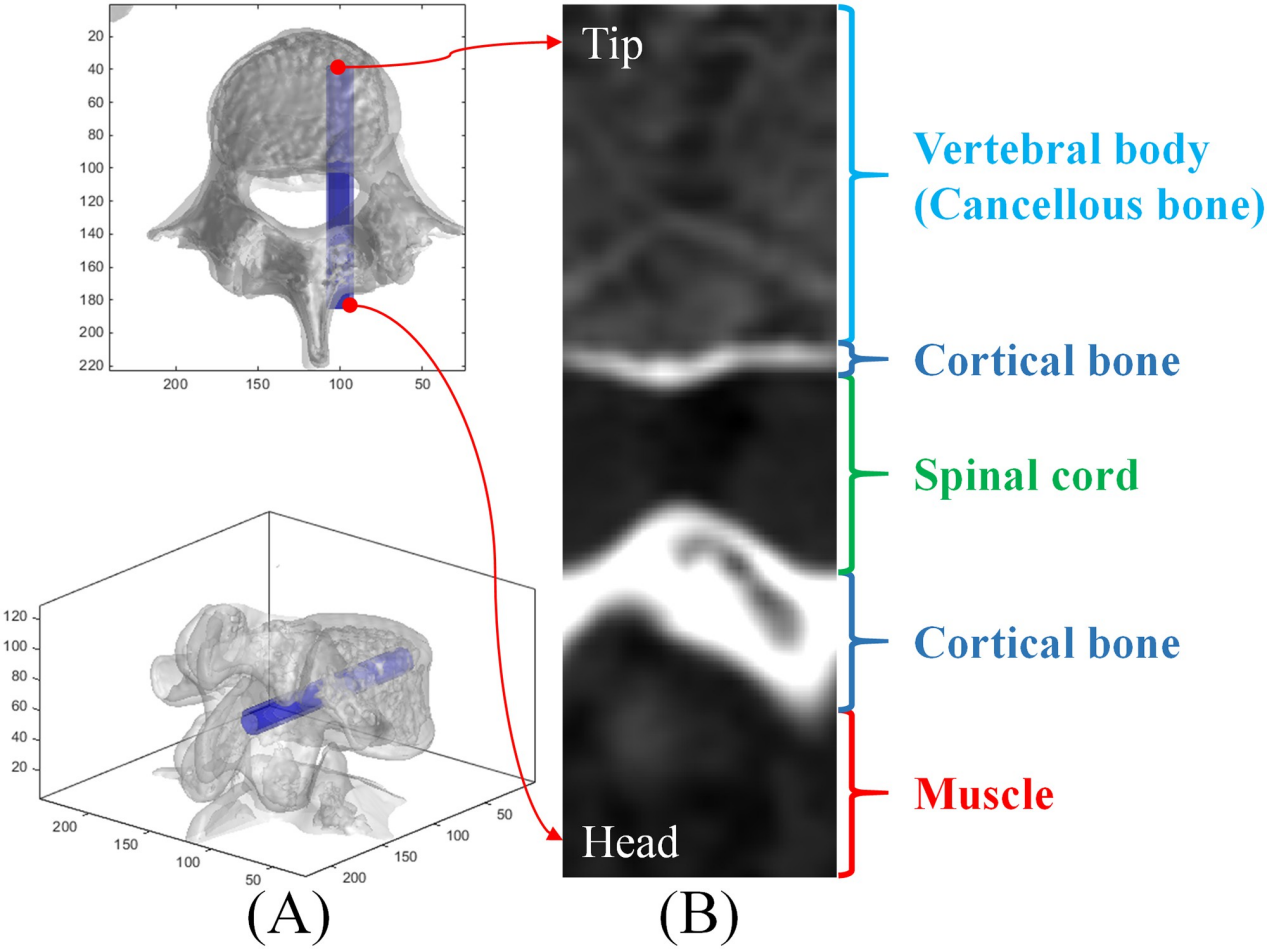
Rod location in the area traversed by a screw and the corresponding reconstructed drilled surface image.

Fig 2B presents the drilled surface image reconstructed using the rods of the area traversed by screws depicted in Fig 2A. Because the grayscale values of drilled surface images, as with typical CT images, were represented in CT number, the grayscale values were similarly interpreted. The drilled surface images clearly displayed the anatomical structure encountered by rods in the described area traversed by screws. This single image aided in the rapid understanding of whether the implantation path was completed within the bone or had pierced through the bone; this assisted in detecting encountered soft tissue, its thickness, and whether any endangered tissues, such as spinal neural tubes, had been encountered.

**Fig 3:**
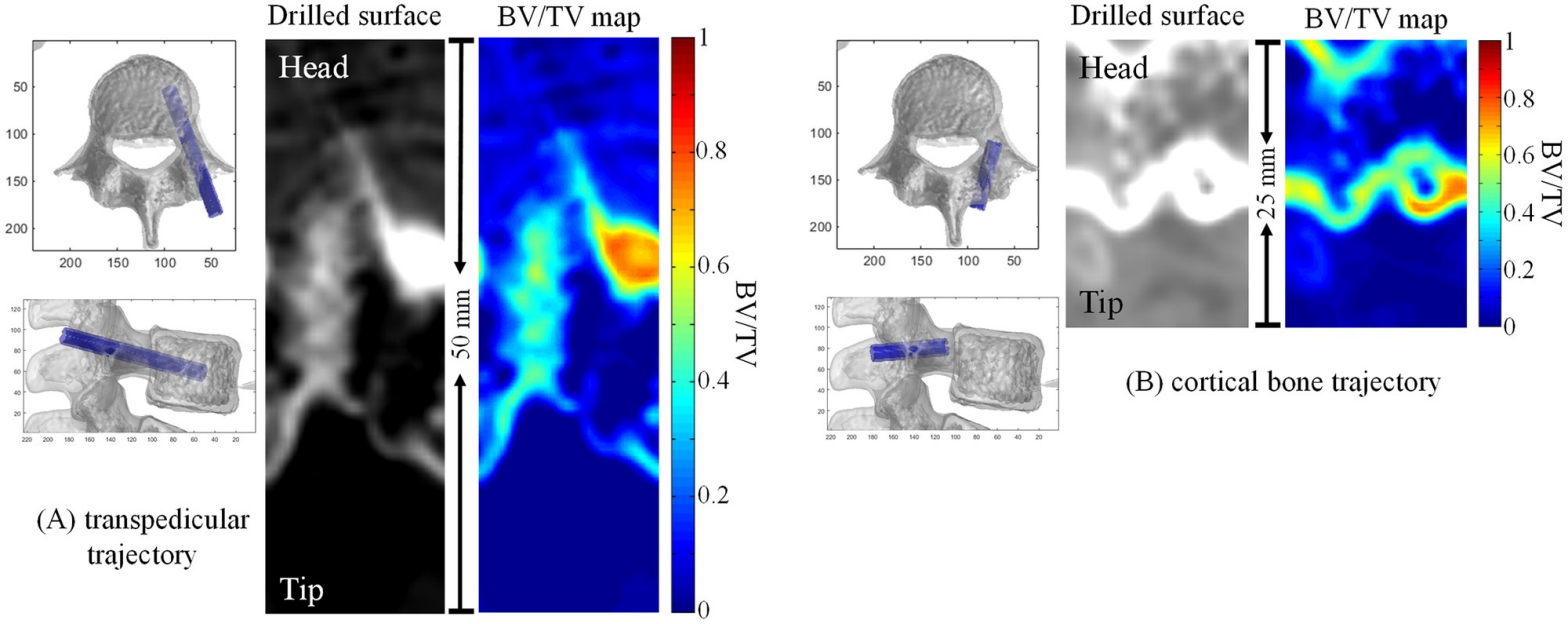
(A) and (B) display drilled surface images reconstructed from rods placed to represent the area traversed by a screw according to the TT and CBT paths in the fourth vertebrae of the same section and the converted CBV map.

Fig 3 displays rods of the described TT and CBT in the fourth vertebrae of the same section, a corresponding reconstructed drilled surface image, and a CBV map. The drilled surface image was used to compare the anatomical exposure of paths, and the CBV map was used in calculations of the total CBV. Furthermore, the drilled surface image directly calculated implantation depth, which facilitated safety assessments of implantation paths and planning. The generated CBV maps directly presented the thickness and proportion of the encountered cortical and spongy bones and indicated areas the bone screws may have encountered in their path.

**Fig 4:**
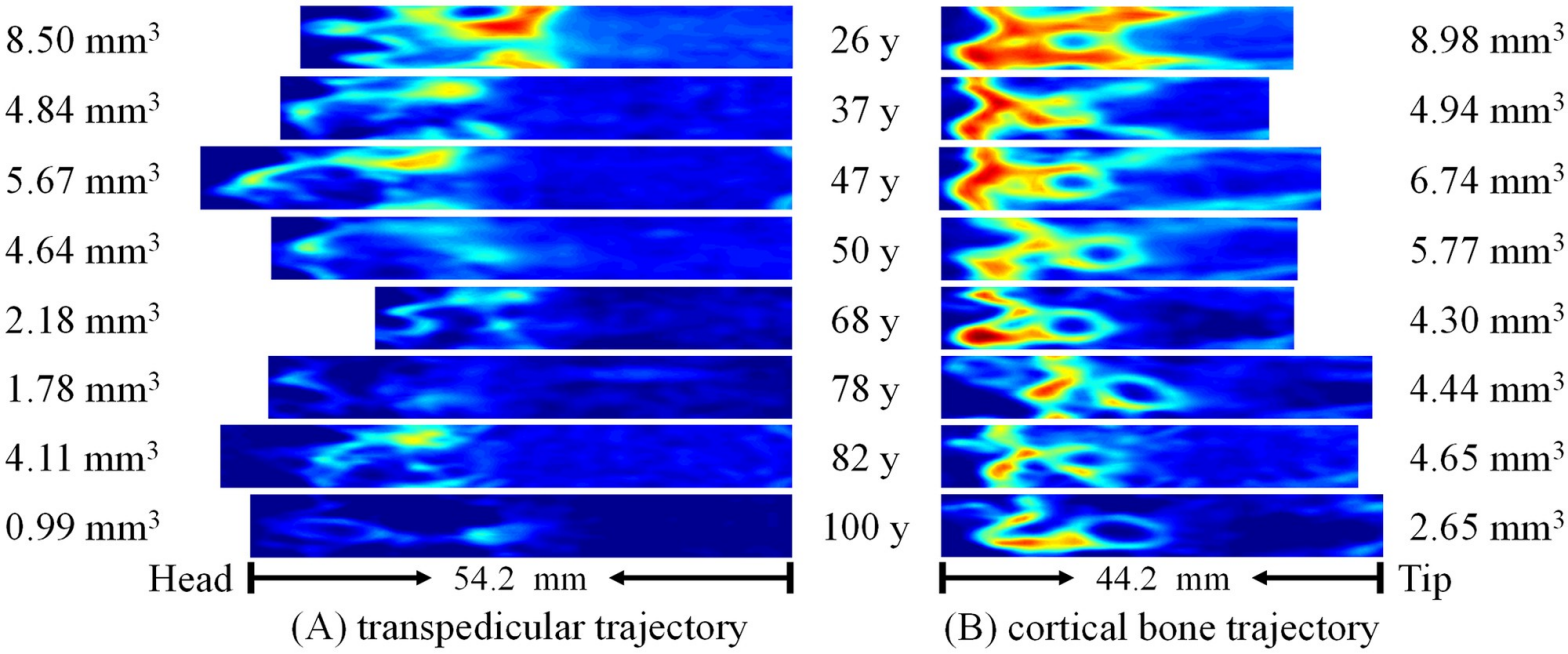
CBV maps reconstructed and converted according the (A) TT and (B) CBT paths on the same side of the fourth vertebrae in patients aged 26–100 years, with the total CBV marked on the map.

Fig 4 presents drilled surface images reconstructed under the TT and CBT paths on the left side of the fourth vertebrae in eight patients aged 26–100 years. The total CBV calculated is marked on the image. These findings indicate that in both TT and CBT paths, the bone volume encountered decreased with age without exception. However, in these eight patients, the total CBV of the CBT path was higher than that of the TT path by 59.9% on average. Among patients aged 50 years and older in particular, the total CBV of the CBT path was higher than that of the TT path by 90.6% on average. Even in a 100-year-old patient, the CBV of the CBT path remained in proximity to that of a 68-year-old patient. Furthermore, the length of drilled surface images represented implantation depth. The CBT implantation length was shorter than the TT length, which suggests that the CBT path may have encountered more bone volume than did the TT path despite its shorter implantation length.

**Fig 5:**
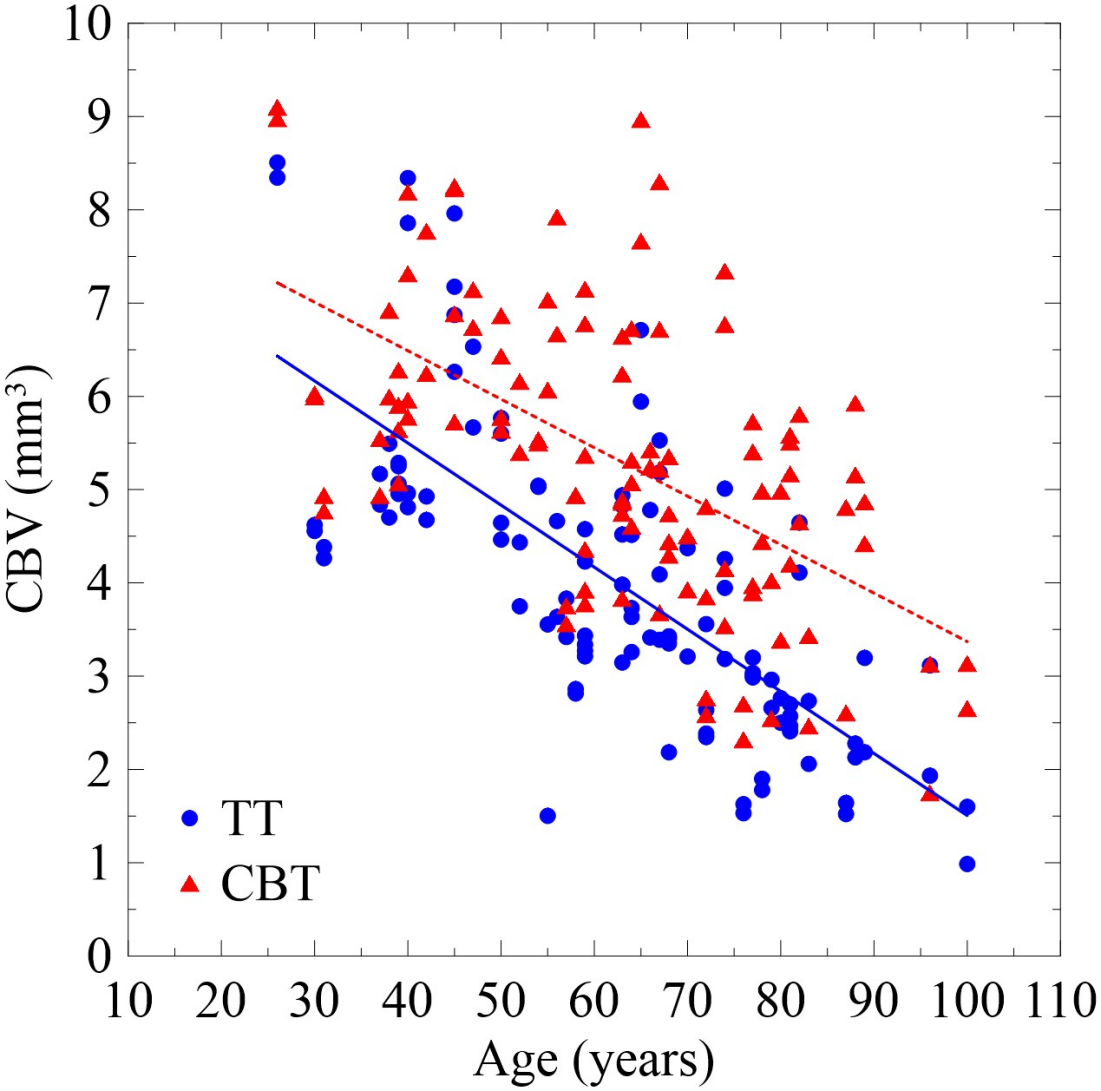
Relationship between age and total CBV in both the TT and CBT paths on both sides of the fourth vertebrae.

**Fig 6:**
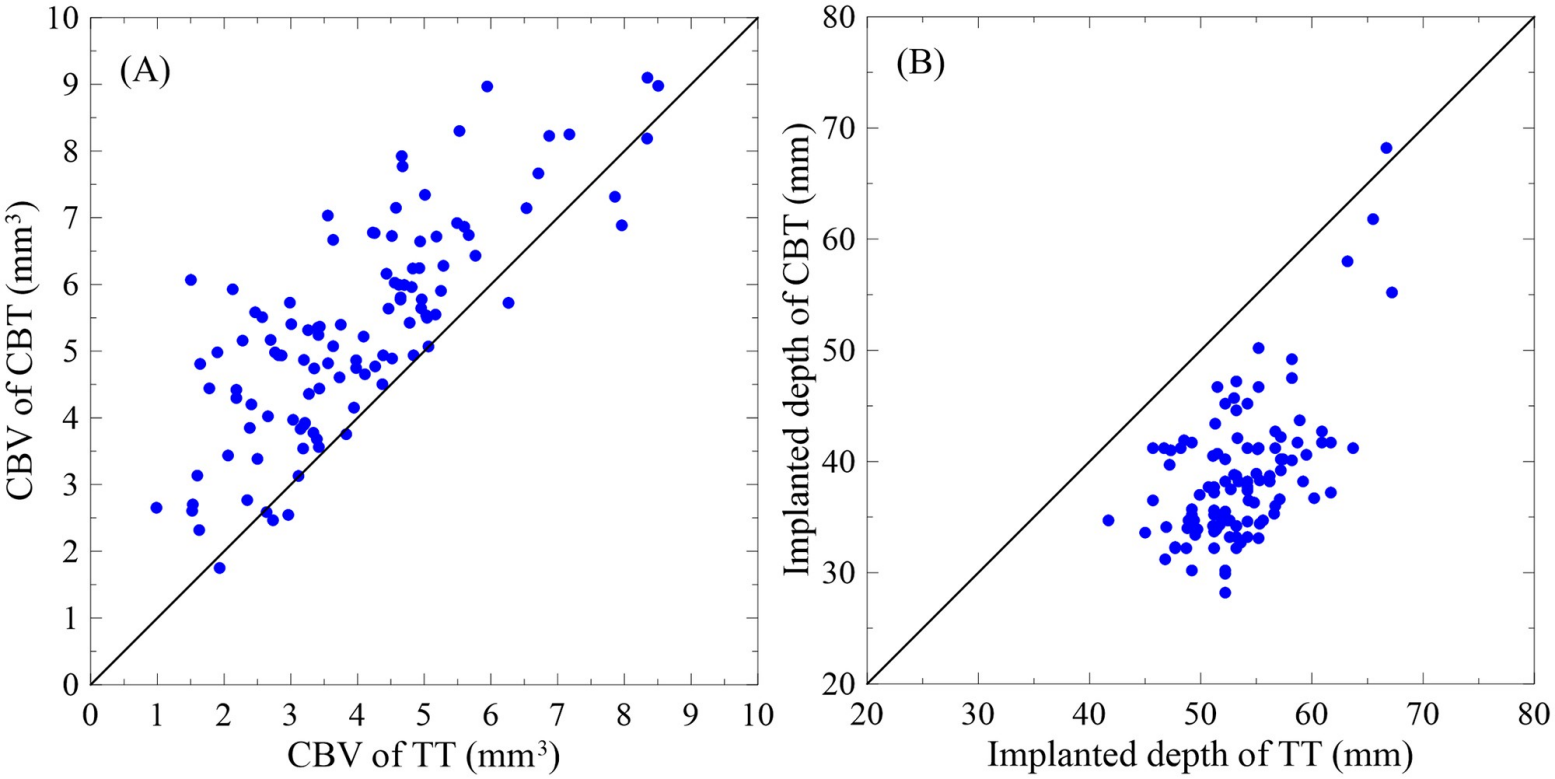
(A) Relationship between total CBV of the TT and CBT paths on the same side of the fourth vertebrae. (B) Relationship between implantation depth of the TT and CBT paths on the same side of the fourth vertebrae.

Fig 5 displays the relationship between age and total CBV in both the TT and CBT paths on both sides of the fourth vertebrae among 55 patients. The total CBV of both paths were observed to decrease with age. Fig 6A presents the relationship between the total CBV of TT and CBT paths on the left side of the fourth vertebrae. The average total CBV of the TT and CBT paths were 5.3 ± 1.6 and 4 ± 1.6 mm^3^, respectively, and the CBT was higher than the TT by approximately 30%. The paired-samples *t*-test revealed a significant difference (*p* < 0.0001), which is consistent with the findings of studies in which CBT exhibited higher resistance than did TT [30, 31]. The correlation coefficient of total CBV and age for the TT and CBT paths were −0.73 and −0.58, respectively (*p*-value < 0.0001), indicating that the correlation between total CBV and age was relatively low in the CBT path compared with the TT path. The *r*^2^ values of the linear fit for the TT and CBT paths were 0.53 and 0.33, respectively. The slopes of the fits for the TT and CBT paths were −0.067 and −0.052, respectively, indicating that the CBV of the CBT path decreased relative slow as age increased compared with the TT path. This was primarily because the CBT path was in the direction of the lamina to the pedicle, and this area mainly comprises cortical bones. The TT path encountered cortical bones only when passing through the pedicle, and after entering the vertebral body, it only encountered the trabeculae within the vertebral body. Unlike the density structure of cortical bones, the trabecula has a sponge-like porous structure, and its density greatly decreases with age and the onset of osteoporosis. The need for spinal fusion surgery is common among patients aged 50 or older with abnormal pressure between vertebrae, and using CBT paths may increase the stability of screws in these patients.

Fig 6B illustrates the relationship between implantation depths for TT and CBT paths on the same side of the fourth vertebrae among 55 patients. The average implantation depth of the TT and CBT paths was 53.5 ± 4.6 and 38.7 ± 6.2 mm, respectively. The CBT paths were shorter than the TT paths by 27.6%. The average CBV of each unit implantation depth for TT and CBT was 0.075 and 0.137 mm^3^/mm, respectively, which demonstrates that CBT cortical implantation strategy encounters many bones at short distances and provides adequate stability.

## Discussion

To elucidate the relationship between implant and anatomical structure, this study proposed reconstructing drilled surface images from cross-sectional images and volumes to overcome the inability of conventional orthographic images to simultaneously observe every part of the implant and anatomical structure. Surface drawings were used to display internal anatomical information. Drilled surface images completely displayed the designated paths of screws in each part and the anatomical structure encountered in one image. Through the use of image quantitative analysis, the inability to effectively assess bone volume screws can be overcome with drilled surface images reconstructed from CT images, which can be converted into CBV maps. This method can be used to evaluate designated paths and compare the stability of paths. The application of this method to compare TT and CBT paths revealed that even when implanted screws were short, CBT effectively increased the contact of screws with bone volume during cortical drilling. For older patients with osteoporosis in particular, the CBV of the TT path was reduced drastically with the disappearance of the trabecular structure in the vertebral body. CBT achieved higher contact bone volume than did TT and ensured higher screw stability.

Reconstructing drilled surface images using CT images can facilitate observation of the contact bone anatomy and subsequent bone volume calculations. The method is not limited to the use of CT images. In path selection, drilled surface images can be reconstructed from magnetic resonance (MR) images for improved visibility of soft tissue and nerve structures to avoid endangering tissue, such as nerves and blood vessels. The reconstruction of drilled surface images can be integrated with intraoperative navigation systems to calculate drilled surface images and CBV in real-time based on direction and previously defined depths designated by surgeons as a reference for implantation. Drilled surface images with CT/MR fusion images can simultaneously aid in the assessment of implantation safety and stability. Furthermore, drilled surface images can be used before surgery to plan and select ideal paths. Their pairing with 3D-printed surgical guides can provide safe and stable implantation [32, 33]. Other implantation procedures, such as pelvis screw surgery, may use drilled surface images to assist in selecting implantation paths.

Because drilled surface images are reconstructed from CT or MR image volumes, image artifacts appearing in original image volumes also appear in the reconstructed drilled surface images, which lowers the diagnostic value of images. Artifacts in CT images lead to errors during later bone volume calculation; therefore, to prevent subsequent errors, image artifacts should be corrected using appropriate methods before reconstructing drilled surface images.

## Conclusion

In this study, a novel drilled surface image reconstructed from designed implanted trajectories of screws and CT image volumes was proposed. These images could completely display the anatomical structure contacted by screws after implantation, which aids in path safety planning. Drilled surface images could assist in calculating contact bone volume in the paths and aided in comparing screw stability across implantation paths. In the study, TT and CBT paths were compared using the proposed method, and when pathological conditions were met, using CBT resulted in higher contact bone volume, which increased implantation stability. Drilled surface imaging can be used in clinical preoperative planning and intraoperative navigation to improve operative quality and safety for clinical spinal fusion surgery.

## Supporting information

S1 Data (PDF)
