# Supplementary material for: Evaluating the contact anatomy and contact bone volume of spinal screws using a novel drilled surface image
Source: PLoS One. 2023 Apr 10;18(4):e0282737. doi: 10.1371/journal.pone.0282737 (PMC10085035; doi:10.1371/journal.pone.0282737)
Supplement: S1 Data — (PDF) [file pone.0282737.s001.pdf]

Patient numbe	Age	Trajectory	Side	Depth (mm)	CBV (mm^3)	Trajectory	Side	Depth (mm)	CBV (mm^3)
1	77	PT	R	55.2	2.99	CBT	R	50.2	5.73
1	77	PT	L	58.2	3.01	CBT	L	47.5	5.40
2	80	PT	R	46.9	2.50	CBT	R	34.1	3.38
2	80	PT	L	45.7	2.76	CBT	L	36.5	4.98
3	63	PT	R	53.2	3.98	CBT	R	38.7	4.86
3	63	PT	L	51.2	3.98	CBT	L	37.2	4.75
4	70	PT	R	52.2	4.37	CBT	R	29.9	4.50
4	70	PT	L	49.2	3.21	CBT	L	30.2	3.92
5	82	PT	R	57.2	4.65	CBT	R	39.2	5.81
5	82	PT	L	49.2	4.11	CBT	L	41.7	4.65
6	74	PT	R	58.2	3.95	CBT	R	49.2	4.15
6	74	PT	L	55.2	3.18	CBT	L	46.7	3.54
7	47	PT	R	60.2	6.53	CBT	R	36.7	7.14
7	47	PT	L	59.2	5.67	CBT	L	38.2	6.74
8	50	PT	R	48.8	4.46	CBT	R	34	5.64
8	50	PT	L	51.3	4.64	CBT	L	35.3	5.77
9	100	PT	R	51.5	1.60	CBT	R	46.7	3.13
9	100	PT	L	54.2	0.99	CBT	L	41.2	2.65
10	39	PT	R	56.2	5.25	CBT	R	38.7	5.90
10	39	PT	L	51.2	4.95	CBT	L	33.7	5.64
11	68	PT	R	47.2	3.40	CBT	R	39.7	5.35
11	68	PT	L	41.7	2.18	CBT	L	34.7	4.30
12	96	PT	R	53.6	3.12	CBT	R	32.7	3.13
12	96	PT	L	61.7	1.93	CBT	L	37.2	1.75
13	89	PT	R	53	3.20	CBT	R	45.7	4.87
13	89	PT	L	48.2	2.18	CBT	L	41.2	4.42
14	72	PT	R	57.2	2.39	CBT	R	42.2	3.85
14	72	PT	L	53.2	3.56	CBT	L	44.6	4.82
15	88	PT	R	49.4	2.28	CBT	R	34.7	5.16
15	88	PT	L	48.9	2.13	CBT	L	34.7	5.93
16	59	PT	R	52.4	3.27	CBT	R	34.7	4.36
16	59	PT	L	55.6	3.44	CBT	L	34.7	5.37
17	55	PT	R	63.2	1.50	CBT	R	58	6.07
17	55	PT	L	67.2	3.55	CBT	L	55.2	7.03
18	78	PT	R	47.3	1.90	CBT	R	41	4.98
18	78	PT	L	51.3	1.78	CBT	L	43.4	4.44
19	37	PT	R	53.2	5.17	CBT	R	34.2	5.55
19	37	PT	L	51.2	4.84	CBT	L	32.2	4.94
20	76	PT	R	52.2	1.53	CBT	R	35.5	2.70
20	76	PT	L	51.7	1.63	CBT	L	34.3	2.32
21	30	PT	R	56.2	4.62	CBT	R	38.2	5.99
21	30	PT	L	52.2	4.56	CBT	L	40.2	6.03
22	42	PT	R	63.7	4.93	CBT	R	41.2	6.24
22	42	PT	L	58.9	4.68	CBT	L	43.7	7.77
23	67	PT	R	57.4	4.09	CBT	R	40.2	5.22
23	67	PT	L	57.2	3.39	CBT	L	40.2	3.68
24	40	PT	R	54.2	4.81	CBT	R	37.4	5.96
24	40	PT	L	54.2	4.96	CBT	L	37.7	5.78
25	26	PT	R	52.2	8.35	CBT	R	38.2	9.10
25	26	PT	L	49.2	8.51	CBT	L	35.2	8.98
26	64	PT	R	58.7	4.52	CBT	R	41.7	6.73
26	64	PT	L	56.7	3.73	CBT	L	41.2	4.61
27	45	PT	R	47.7	7.18	CBT	R	32.2	8.25
27	45	PT	L	48.7	6.87	CBT	L	32.2	8.23
28	31	PT	R	53.2	4.26	CBT	R	32.2	4.77
28	31	PT	L	54.2	4.38	CBT	L	33.2	4.94
29	87	PT	R	52.2	1.64	CBT	R	45.2	4.81
29	87	PT	L	53.2	1.52	CBT	L	47.2	2.61
30	65	PT	R	66.7	5.94	CBT	R	68.2	8.97
30	65	PT	L	65.5	6.71	CBT	L	61.8	7.66
31	40	PT	R	53.4	8.34	CBT	R	38.2	8.19
31	40	PT	L	51.2	7.86	CBT	L	35.2	7.31
32	68	PT	R	54.2	3.35	CBT	R	45.2	4.74
32	68	PT	L	56.7	3.43	CBT	L	42.7	4.44
33	63	PT	R	45.7	3.15	CBT	R	41.2	3.83
33	63	PT	L	46.7	4.52	CBT	L	41.2	4.89
34	79	PT	R	58.2	2.96	CBT	R	40.1	2.55
34	79	PT	L	54.2	2.66	CBT	L	38.2	4.02
35	77	PT	R	55	3.03	CBT	R	38.9	3.97
35	77	PT	L	51.2	3.20	CBT	L	37.7	3.89

36	45	PT	R	61.7	6.26	CBT	R	41.7	5.72
36	45	PT	L	60.9	7.96	CBT	L	41.7	6.89
37	39	PT	R	52.2	5.29	CBT	R	30.2	6.28
37	39	PT	L	52.2	5.06	CBT	L	28.2	5.07
38	38	PT	R	55.2	5.49	CBT	R	41.2	6.92
38	38	PT	L	49.2	4.70	CBT	L	35.7	5.99
39	59	PT	R	51.1	3.34	CBT	R	34.2	3.78
39	59	PT	L	53.2	3.21	CBT	L	34.2	3.92
40	64	PT	R	52.6	3.26	CBT	R	34.7	5.31
40	64	PT	L	53.2	3.63	CBT	L	33.2	5.07
41	81	PT	R	48.5	2.47	CBT	R	41.9	5.58
41	81	PT	L	51.1	2.70	CBT	L	40.5	5.17
42	54	PT	R	57.1	5.03	CBT	R	36.6	5.54
42	54	PT	L	56.7	5.04	CBT	L	36	5.50
43	58	PT	R	51.6	2.81	CBT	R	35.2	4.94
43	58	PT	L	52.7	2.86	CBT	L	37.5	4.94
44	52	PT	R	54.3	3.75	CBT	R	36.5	5.40
44	52	PT	L	55.3	4.43	CBT	L	34.4	6.16
45	72	PT	R	47.7	2.64	CBT	R	32.3	2.59
45	72	PT	L	46.8	2.35	CBT	L	31.2	2.77
46	56	PT	R	56.6	3.63	CBT	R	35.3	6.67
46	56	PT	L	54.8	4.66	CBT	L	36.3	7.92
47	57	PT	R	55.2	3.42	CBT	R	33.1	3.56
47	57	PT	L	54.2	3.83	CBT	L	34.6	3.75
48	74	PT	R	49.9	5.01	CBT	R	37	7.34
48	74	PT	L	53	4.26	CBT	L	38.8	6.77
49	81	PT	R	51.5	2.57	CBT	R	40.7	5.51
49	81	PT	L	51.2	2.41	CBT	L	35.6	4.20
50	59	PT	R	55.3	4.23	CBT	R	38.3	6.78
50	59	PT	L	55.2	4.58	CBT	L	41.2	7.15
51	66	PT	R	49.7	4.78	CBT	R	33.9	5.42
51	66	PT	L	45	3.41	CBT	L	33.6	5.24
52	67	PT	R	50.7	5.53	CBT	R	37.7	8.30
52	67	PT	L	51.4	5.18	CBT	L	33.9	6.72
53	63	PT	R	55.1	4.94	CBT	R	41.1	6.64
53	63	PT	L	53.3	4.83	CBT	L	42.1	6.24
54	83	PT	R	49.5	2.06	CBT	R	33.4	3.44
54	83	PT	L	52.6	2.73	CBT	L	33.2	2.46
55	50	PT	R	59.5	5.60	CBT	R	40.6	6.86
55	50	PT	L	60.9	5.77	CBT	L	42.7	6.43
